# Expanding molecular diagnostics of helminthiasis: Piloting use of the GPLN platform for surveillance of soil transmitted helminthiasis and schistosomiasis in Ghana

**DOI:** 10.1371/journal.pntd.0006129

**Published:** 2018-01-25

**Authors:** Lucas J. Cunningham, John Odoom, Deborah Pratt, Linda Boatemaa, Nana Asante-Ntim, Keren Attiku, Bismarck Banahene, Mike Osei-Atweneboana, Jaco J. Verweij, David Molyneux, Russell J. Stothard, Emily R. Adams

**Affiliations:** 1 Parasitology Department, Liverpool School of Tropical Medicine, Liverpool, United Kingdom; 2 Virology Department, Noguchi Memorial Institute for Medical Research, Accra, Ghana; 3 Department of Environmental Biology and Health, Council for Scientific and Industrial Research, Accra, Ghana; 4 Laboratory for Medical Microbiology and Immunology, St Elisabeth Hospital, Tilburg, the Netherlands; Baylor College of Medicine, UNITED STATES

## Abstract

The efforts to control and eradicate polio as a global health burden have been successful to the point where currently only three countries now report endemic polio, and the number of cases of polio continues to decrease. The success of the polio programme has been dependant on a well-developed network of laboratories termed the global polio laboratory network (GPLN). Here we explore collaborative opportunities with the GPLN to target two of the 18 diseases listed as a neglected tropical diseases (NTD) namely soil transmitted helminthiasis (STH) and *Schistosomiasis* (SCH). These were chosen based on prevalence and the use of faecal materials to identify both polio, STH and SCH. Our study screened 448 faecal samples from the Ghana GPLN using three triplex TaqMan assays to identify *Ascaris lumbricoides*, *Necator americanus*, *Ancylostoma spp*, *Trichuris trchiura*, *Strongyloides stercoralis* and *Schistosoma* spp. Our results found a combined helminth prevalence of 22%. The most common helminth infection was *A*. *lumbricoides* with a prevalence of 15% followed by *N*. *americanus* (5%), *Ancylostoma* spp. (2.5%), *Schistosoma* spp. (1.6%) and *S*. *stercoralis* (1%). These results show that it is possible to identify alternative pathogens to polio in the samples collected by the GPLN platform and to introduce new diagnostic assays to their laboratories. The diagnostic methods employed were also able to identify *S*. *stercoralis* positive samples, which are difficult to identify using parasitological methods such as Kato-Katz. This study raises the possibility of collaboration with the GPLN for the surveillance of a wider range of diseases which would both benefit the efforts to control the NTDs and also increase the scope of the GPLN as a diagnostic platform.

## Author summary

The successful campaign being waged against polio has eliminated the disease from most countries where it was once endemic. With this success, it is anticipated that the disease will be eradicated in the coming years with only 37 cases being reported in 2016. Although the efforts to control polio are successful there are a number of low-profile, but no less serious disease, that are still highly prevalent throughout the world. These diseases have been termed the neglected tropical diseases (NTD) and this study aims to test the suitability of the Global Polio Laboratory Network (GPLN) as a platform to screen for two of the NTDs, soil transmitted helminthiasis (STH) and schistosomiasis (SCH). To test the suitability of the samples collected by the GPLN and the suitability of the laboratories themselves 448 samples from the Ghanaian GPLN laboratory were screened with multiplex TaqMan assays for the following six helminth types: *Ascaris lumbricoides*, *Necator americanus*, *Ancylostoma spp*, *Trichuris trchiura*, *Strongyloides stercoralis* and *Schistosoma* spp. Using this method this study was able to identify a prevalence of 22% for the combined helminth infection. The most common infection was *A*. *lumbricoides* with a prevalence of 15% followed by *N*. *americanus* (5%), *Ancylostoma* spp. (2.5%), *Schistosoma* spp. (1.6%) and *S*. *stercoralis* (1%). The success of this study indicates that this may be a cost-effective method to passively screen a country for STH and SCH and its success in identifying *S*. *stercoralis* infections makes it especially useful as this parasite is hard to identify using traditional surveillance techniques.

## Introduction

In 1988 the WHO set out to eradicate polio after the successful development of effective polio vaccines and since then the eradication campaign has reduced the number of countries reporting endemic polio from 125 to three in 2016. Control of polio has been a co-ordinated effort involving two main arms; the delivery of vaccination alongside establishing an effective laboratory network for monitoring and surveillance. This surveillance arm is comprised of 145 labs spread throughout the world which taken as a whole forms the Global Polio Laboratory Network (GPLN). The network receives samples from local health clinics where individuals have presented with clinical signs of the disease, typically acute flaccid paralysis (AFP), with the need to confirm or exclude an aetiology of polio. Thus, investigation of AFP initially involves collection of a faecal sample(s) which is then transferred from the regional clinic thence to the central laboratory to undergo a culture screen for polio virus and if found positive is then followed up with a real-time PCR analysis with diagnostic primers able to identify and discriminate if the sample is wild type, vaccine strain or a vaccine-derived virus. Across Africa there are 16 GPLN labs and these have received, in total, an average of 22,017 samples per year in the past 5 years with Ghana contributing, on average, 350 samples per year. These 16 laboratories are divided into three regional reference laboratories (RRLs) and 13 intratypic differentiation laboratories (ITD). The ITD laboratories are responsible for the isolation of poliovirus, molecular characterization of isolates and referral of critical samples to a sequencing laboratory [1]. Currently faecal samples collected by the GPLN are only screened for polio and non-polio enteroviruses; here we explore the potential of the GPLN to screen for other pathogens of public health importance allowing for co-investigation.

Across the world, but especially in Africa, the Neglected Tropical Diseases (NTDs) are an umbrella group of diseases that afflict the poor and retain a cycle of poverty. In total, approximately a billion people from the poorest communities across the globe are infected with at least one NTD [2]. There are currently 18 diseases listed as NTDs [3] with seven of these diseases caused by parasitic helminths. The intestinal nematodes, often referred to as soil transmitted helminths (STH) contribute the greatest number of infections and highest number of DALYs lost for any NTD [4] closely followed by schistosomiasis (SCH), a waterborne trematode infection [5]. Recently the importance of control of these diseases has been recognised by policy makers and steps have been taken to develop cost effective strategies in managing them [6]. Although there are WHO guidelines for classic parasitological surveillance, there is no equivalent for a molecular diagnostic platform of these infections. A key block in doing so is the cost of setting up a standard surveillance platform, *de novo*, it would therefore be sensible to expand and strengthen existing surveillance structures. The GPLN is a good example and is maintained with substantial annual investments [7]. Thus being able to augment or ‘piggyback’ appropriate NTD surveillance onto the GPLN could have the necessary ‘kick-starting’ effect to provide better access to diagnostic tools needed for control and elimination of STH and SCH [8–12].

Addressing the need for a molecular diagnostics platform for NTDs, in this investigation we explore and develop synergies and necessary steps with the GPLN, taking advantage of its accumulated experience and resources, to include pilot screening for STH (*A*. *lumbricoides*, *N*. *americanus*, *Ancylostoma spp*, *T*. *trchiura*, *S*. *stercoralis*) and SCH (*S*. *mansoni* and *S*. *haematobium*). The Ghanaian National Polio laboratory based at the Noguchi Memorial Institute for Medical Research was selected to carry out this assessment determining the suitability of the GPLN faecal collections with multiplex TaqMan diagnostic assays.

## Methods

### Ethics

Ethics applications were approved by LSTM (Research protocol 16-007) and Noguchi Scientific and Technical Committee (Study number 065/16-17) followed by the Institutional Review Board. To obtain approval, initial patient collection forms were amended to later facilitate expanded diagnostic testing with the results made available to the national NTD programme. All participants were anonymised for the final study.

### Training workshop

Prior to the work being carried at the Ghanaian GPLN a workshop was carried out to train the staff in the methods used to extract DNA from faecal samples and the subsequent optimisation and running of the qPCR TaqMan assays. The workshop included both practical and theoretical training, this gave the staff of the GPLN a good background knowledge and practical experience in the methods they would use [13, 14].

### Samples

The samples used in this study were faecal samples sent to the Ghanaian GPLN laboratory from individuals presenting with acute flaccid paralysis. The samples were sent via courier from local health clinics and were kept at 4°C until it reached the GPLN laboratory at which point they were stored at -20°C. The age of the patients that supplied the sample as well as the district from which it originated from were available to this study.

### DNA extraction

Faecal samples were removed from the -20°C freezer and allowed to defrost at room temperature, once defrosted ~0.1g of faeces was removed and placed into a 2mL screw cap sample tube that was preloaded with 0.9g of 1.4mm ceramic beads. To this 250μL of a 2% PVPP/PBS suspension was added and the sample vortexed for 5-10 seconds. The faecal suspension was then frozen at -20°C overnight. The following day the samples underwent bead-beating at 3000rpm for 30 seconds using the MagnaLyser system. DNA extraction was carried out using the QIAamp DNA Mini kit per the manufacturer’s instructions with the following two modifications: i) an aliquot of phocine herpes virus-1 was added to the AL buffer to act as an internal positive control for the subsequent TaqMan assays, ii) the DNA was eluted in 200μL of nuclease free water. As well as introducing Phocine Herpes Virus (PhHV) into each sample to act as an internal positive control a DNA extraction negative control was introduced after every 47^th^ sample, in total 448 samples were processed [15].

### Multiplex qPCR

The six helminth types were screened using previously described primers and probes [8–10, 12, 16, 17] and these were used in three triplex reactions, each targeting two helminth types and the internal positive control. The first of these targeted *S*. *stercoralis* and *N*. *americanus*; the second targeted *Ancylostoma* spp. and a generic *Schistosoma* spp. (*S*. *mansoni*, *S*. *haematobium*, *S*. *intercalatum*); the third triplex reaction targeted *A*. *lumbricoides* and *T*. *trichiura* (Table 1). The primer concentrations were determined individually through primer limiting assays and then tested in the final triplex concentrations using mono, double and triple target DNA assays to ensure there was no internal competition within a reaction. The final volume for each triplex reaction was 20μL, consisting of 12.5μL of iQ supermix, 2μL of DNA template and a final helminth primer concentration of 200nM except for *Ancylostoma* spp. which ran at 300nM; the concentration of all probes and the PhHV primers was 100nM. All assays were processed using the same ABI 7500 qPCR thermocycler. Each qPCR run consisted of the following cycle, an initial holding step at 95°C for three minutes followed by 50 cycles of 95°C for 15s, 60°C for 30s, 72°C for 30s and a final extension step at 72°C for two minutes.

**Table 1:** Primer and probes used in this study.

Multiplex	Probe/Primer	Target	Probe and Primer sequence (5'-3')	Described
1	Forward	*S*. *stercoralis*	GAA TTC CAA GTA AAC GTA AGT CAT TAG C	Verweij 2009
	Reverse		TGC CTC TGG ATA TTG CTC AGT TC	
	Probe		FAM-ACA CAC CGG CCG TCG CTG C-BHQ1	
	Forward	*N*. *americanus*	CTG TTT GTC GAA CGG TAC TTG C	Verweij 2007
	Reverse		ATA ACA GCG TGC ACA TGT TGC	
	Probe		TAMRA-CTG TAC GCA TTG TAT AC-BHQ2	
2	Forward	*Ancylostoma* spp.	GAA TGA CAG CAA ACT CGT TGT TG	Verweij 2007
	Reverse		ATA CTA GCC ACT GCC GAA ACG T	
	Probe		TAMRA-ATC GTT TAC CGA CTT TAG-BHQ2	
	Forward	*Schistosoma* spp.	GGTCTAGATGACTTGATYGAGATGCT	Obeng 2008
	Reverse		TCCCGAGCGYGTATAATGTCATTA	
	Probe		FAM-TGG GTT GTG CTC GAG TCG TGG C-BHQ1	
3	Forward	*A*. *lumbricoides*	GTA ATA GCA GTC GGC GGT TTC TT	Wiria 2010
	Reverse		GCC CAA CAT GCC ACC TAT TC	
	Probe		TAMRA-TTG GCG GAC AAT TGC ATG CGA T-BHQ2	
	Forward	*T*. *trichiura*	TTGAAACGACTTGCTCATCAACTT	Liu 2013
	Reverse		CTGATTCTCCGTTAACCGTTGTC	
	Probe		FAM-CGA TGG TAC GCT ACG TGC TTA CCA TGG-BHQ1	
1,2,3	Forward	Phocine herpes virus	GGG CGA ATC ACA GAT TGA ATC	Niesters 2002
	Reverse		GCG GTT CCA AAC GTA CCA A	
	Probe		VIC-TTT TTA TGT GTC CGC CAC CAT CTG GAT C-BHQ2	

## Results

### Location

The 10 regions of Ghana were used to categories the origins of the samples collected by the Ghanaian GPLN. This was then used to determine how evenly across Ghana the origins of the samples were distributed. The region that contributed the most samples was Brong Ahafo, where 23% of the samples originated from following this was the Western region, supplying 15% of the samples. The Ashanti, Central, Greater Accra, Northern, Upper East and Volta regions all contributed a similar amount of between 7-10%. The regions that contributed the least number of samples were the Eastern and Upper West Regions. The distribution of the participants that supplied the samples can be shown to have come from across the country with most regions contributing a similar number of samples.

### Age

Due to the anonymity of the samples the sex of the participants was unknown and could not be included as a risk factor, however their age was recorded. It was possible to observe the age range of samples as this would affect the suitability of samples for STH screening. Across the 10 regions the average age ranged from 5 to 6 years and an analysis using ANOVA resulted in a P = 0.16, indicating there was no significant difference in participants age across the six regions of Ghana. Breaking down the ages of participants into pre-school age (PSAC, 0-4 yrs), school age (SAC, 5-16yrs) and adults (17+) the following percentages were found for each group: 60%, 30% and 10% respectively.

### Parasitology

The qPCR assay was successful in identifying positive samples for *A*. *lumbricoides*, *N*. *americanus*, *A*. *duodenale*, *Schistosoma* and *S*. *stercoralis*. A total of 102 out of 448 samples were found to be positive for one or more helminth types tested, giving an overall prevalence of 22.7% with 92 of these being single helminth infections and 10 being double infections (Table 2)

**Table 2:** Species contribution to single and mixed helminth infections.

Single Infections						
*Ascaris*	*Necator*	*Ancylostoma*	*Trichuris*	*Schistosoma*	*Strongyloides*	Total
59	15	10	0	5	3	92
Double Infections						
*Ascaris/Necator*	*Asccaris/Schistosoma*	*Ascaris/Strongyloides*		*Necator /Ancylostoma*		Total	
5	2	2		1		10	

**Fig 1:**
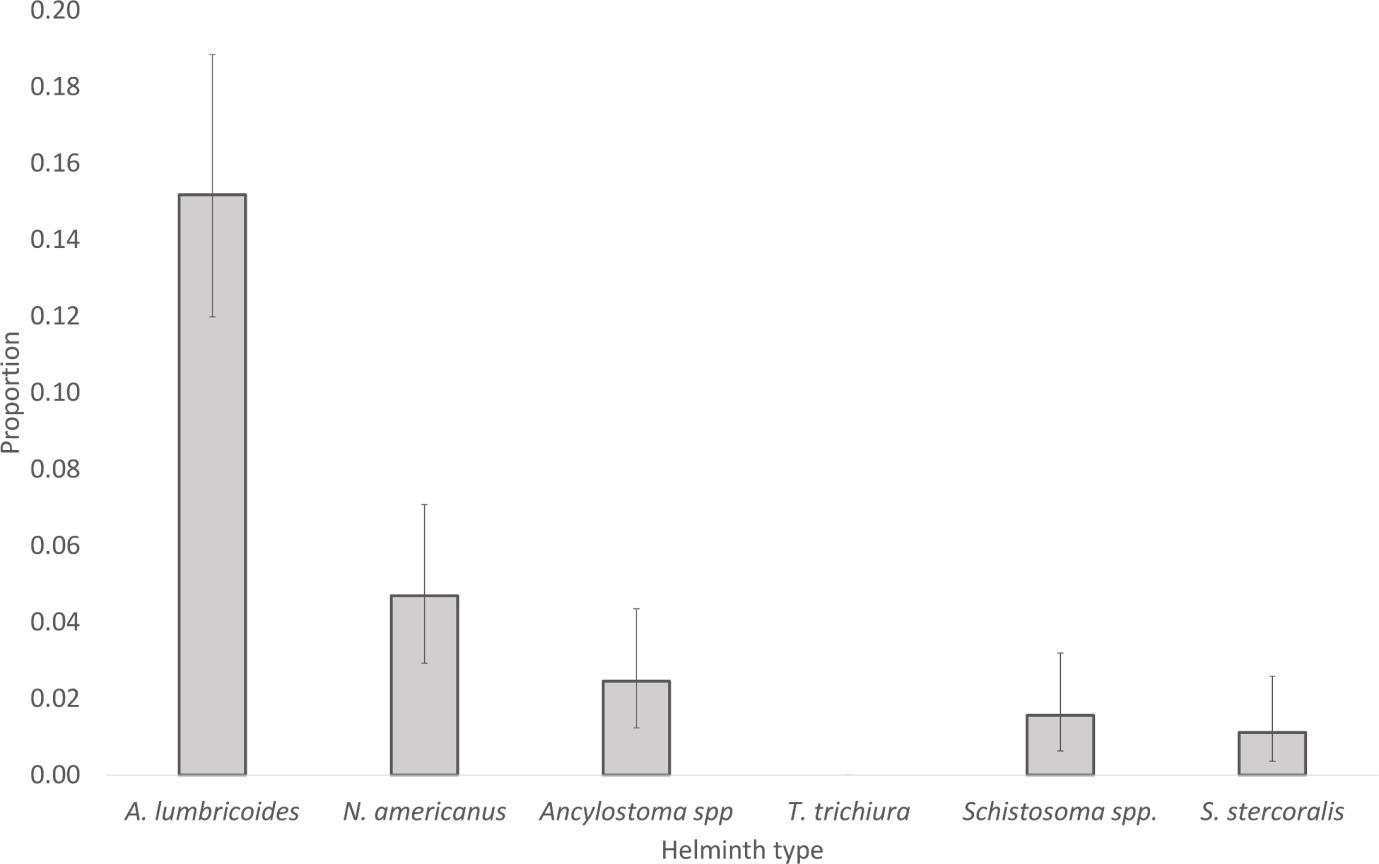
Proportion of helminth types found.

The proportion of samples positive for the different helminth types is shown in Fig 1, *A*. *lumbricoides* was found to be the most prevalent helminth, being found in 16% of all samples. The two-hookworm species followed with *N*. *americanus* found in 6% of samples and *Ancylostoma spp*. found in 3%. The prevalence of *Schistosoma* spp. and *S*. *stercoralis* was 2% and 1% respectively whilst no samples were found to be positive for *T*. *trichiura*.

**Fig 2:**
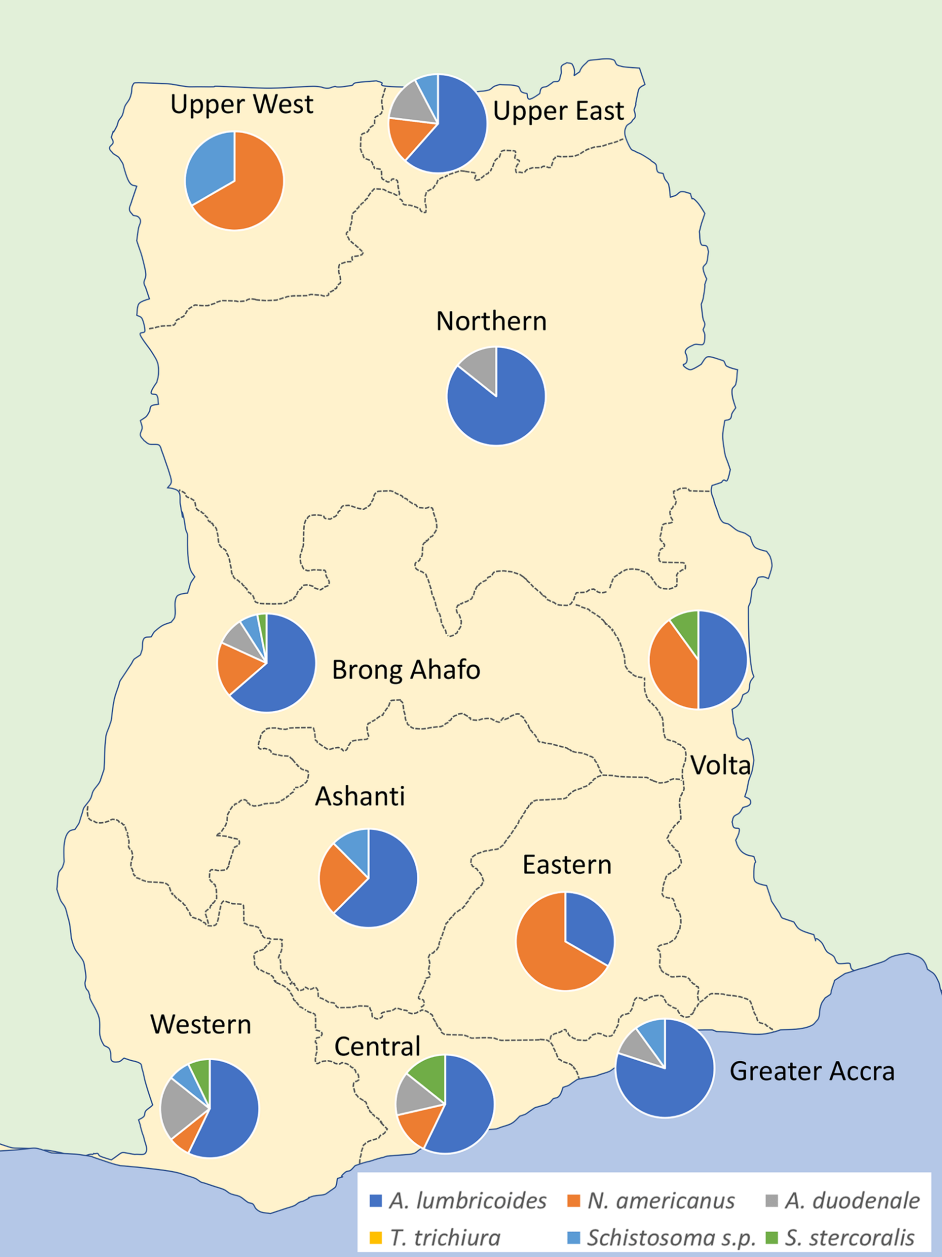
Geographical distribution of the different helminth positives from the GPLN samples screened in this study, Image created using GNU Image Manipulation Program [18].

The distribution of helminth species across the different regions of Ghana varied with Brong Ahafo having the highest proportion of positive samples and the central region having the lowest proportion of positives. The distribution of helminth species across these regions was also not even with the Upper West, Northern and Eastern regions only being positive for two of the helminth types (Fig 2). Other regions contained samples positive for multiple species of helminth.

## Discussion

The purpose of this study is to demonstrate the suitability of adapting a GPLN laboratory for the detection of STH and SCH. It is not within the scope of this study to infer anything from the epidemiological data as the sample size is too small to be representative of the different administrative regions of Ghana. Similarly the samples collected by the GPLN will not be representative of the communities they originate from as they are from individuals that have presented with specific clinical symptoms, notably acute flaccid paralysis.

There were a total of 102 helminth positive samples detected out of a total of 448 samples screened, of which 92 were single infections comprising *A*. *lumbricoides* (59), *N*. *americanus* (15) and *Ancylostoma* spp. (10) respectively. *Schistosoma* spp. (5) was the next most common helminth although surprisingly no cases of *Schistosoma* spp. were detected in samples from the Volta. *S*. *stercoralis* is perhaps the least understood of the intestinal helminths [19] and is difficult to detect with traditional techniques, despite this our study was able to identify five cases of *S*. *stercoralis*, three single infections and two co-infections with *A*. *lumbricoides*. The total number of samples positive for co-infections was 10 of which half were a co-infection of *A*. *lumbricoides* and *N*. *americanus*.

The results show that the average age of participants falls between five to six years which means they fall within the SAC age group which is the usual target group for STH and SCH prevalence surveys. The sample contribution from each region varied from 23% to 3% however seven out of the 10 regions contributed a similar percentage of samples. Surprisingly no SCH positives were found in samples from the Volta. The reason for the lack of SCH positives from the Volta region is not yet clear and could be due to insufficient samples from this region, although this is unlikely as regions contributing fewer samples were still found to have SCH positives. An alternative explanation for the lack of *Schistosoma* s.l. positives is that the method described in this paper is more suited to the detection of *S*. *mansoni* than it is for *S*. *haematobium*, whose eggs are typically passed in stool samples, whereas *S*. *haematobium* predominantly pass their eggs in urine [20].

The scientific community has long acknowledged the likely high burden of disease and morbidity that is caused by *S*. *stercoralis* [21]. Current estimates of global infection range from 30 to 100 million [22, 23] although these estimates are based on imperfect sampling techniques and a more recent study has proposed a higher prevalence of 370 million people infected world-wide [24]. This wide range in prevalence estimates highlights the variable reliability of different screening methods. The most common method of screening for helminth infections, Kato-Katz, is poorly suited to the detection of *S*. *stercoralis* [25]. The success of the methodology used in this paper to detect *S*. *stercoralis* alongside the other STH species and *Schistosoma* spp. demonstrate the versatility of using qPCR to detect a wider range of helminth infections than more traditional methods. Currently there is no data regarding the distribution of *S*. *stercoralis* in Ghana however by screening the samples from the GPLN we were able to identify five samples positive for *S*. *stercoralis*.

The current design of the GPLN is not yet suited for its samples to be used to infer the distribution of STH and SCH as the samples are too few in number and are from a specific group within the population, those presenting with clinical signs of polio. The introduces confounding factors and does not provide a representative cross section of the communities at risk, notably adults and school age children were fewer in number than pre-school age children. To become an adequate surveillance platform the range of clinical symptoms for sample collection would need to be widened to include those presenting with STH and SCH symptoms. This would no doubt increase the number of samples being sent in for analysis and subsequently improve the surveillance capabilities of the system. However, this would no doubt incur a greater cost, a possible solution would be to incorporate other pathogens for screening to attract extra funding to cover these costs.

In conclusion, the findings of this study show that it is possible to identify STH and SCH positives in the faecal samples collected by the GPLN and that new diagnostic techniques can be introduced to compliment the work currently being carried out. The current narrow clinical symptoms required to qualify a sample to be sent to the GPLN limits their epidemiological use, a change in sample submission policy would be required to improve their epidemiological relevance. Despite this the study demonstrates a potential way forward in the monitoring and control of NTDs that could be included in the legacy plan of the GPLN.
